# Real-time Crystal Growth Visualization and Quantification by Energy-Resolved Neutron Imaging

**DOI:** 10.1038/srep46275

**Published:** 2017-04-20

**Authors:** Anton S. Tremsin, Didier Perrodin, Adrian S. Losko, Sven C. Vogel, Mark A.M. Bourke, Gregory A. Bizarri, Edith D. Bourret

**Affiliations:** 1 Space Sciences Laboratory, University of California at Berkeley, Berkeley, CA 94720, USA; 2 Lawrence Berkeley National Laboratory, Berkeley, CA 94720, USA; 3 MST-8, Los Alamos National Laboratory, Los Alamos, NM 87545, USA; 4 P-DO, Los Alamos National Laboratory, Los Alamos, NM 87545, USA

## Abstract

Energy-resolved neutron imaging is investigated as a real-time diagnostic tool for visualization and *in-situ* measurements of “blind” processes. This technique is demonstrated for the Bridgman-type crystal growth enabling remote and direct measurements of growth parameters crucial for process optimization. The location and shape of the interface between liquid and solid phases are monitored in real-time, concurrently with the measurement of elemental distribution within the growth volume and with the identification of structural features with a ~100 μm spatial resolution. Such diagnostics can substantially reduce the development time between exploratory small scale growth of new materials and their subsequent commercial production. This technique is widely applicable and is not limited to crystal growth processes.

Single crystals are core elements in electronic, optical, microwave, and numerous other devices in domains as diverse as medical imaging, security screening, high energy physics, space exploration and many others. Key to any viable development of these applications are yield, reproducibility and final cost of the material production. The optimization of the latter for large size industrial scale has always been challenging. A new crystal material and its growth technique almost always require specific time consuming optimization as limited knowledge can be inferred from previous developments. *In situ* diagnostics combined with computer simulation could substantially decrease the process development time and provide guidance for real-time feedback control and optimization of crystal growth. However, such diagnostics are difficult to implement. For example, the Bridgman crystal growth process^1^,^2^, developed in 1925, is a widely used technique, due to its relative simplicity, both for the discovery studies of new materials in single crystal form and in large-scale production^3^. However, the Bridgman technique is effectively a “blind” crystal growth method, as little information on the crystal can be obtained in real time. In some cases transparent furnaces that allow direct view of the growth vessel can be used for Bridgman growth. They can provide a global view of the crystal growing only for non-refractory materials, with no volumetric images with quantitative information. Therefore the exact location and shape of the interface between the liquid and solid phases, its elemental composition, the temperature and defects distribution within the material, and other parameters in many cases can be estimated only through an indirect measurement on the periphery of the growth volume or through post-growth characterization as no probes can be introduced directly into the growing materials, resulting in months of trial and error experiments. In addition, simulations of crystal growth, which are widely used for process optimization^4^,^5^,^6^, have large uncertainties due to the lack of sufficient information on physical properties of new materials. This makes it difficult to validate the simulation and to ensure that the model is representative of the actual growth process.

Real-time neutron imaging technique appears to be a unique tool for *in situ* characterization of the crystal growth process. It also provides support for the validation of simulations. Neutrons can penetrate the thermal insulation and structural materials used in most pullers, i.e. Bridgman furnaces, and probe the growth volume through neutron absorption and scattering. The attenuation cross section for neutrons in general is not proportional to the atomic number of a particular element, as in the case of X-rays, because neutrons interact, except for magnetic scattering, with the nucleus of the atom and not with its electrons. Therefore, many materials opaque to conventional X-ray techniques can still be interrogated with neutrons. This approach enables multimodality provided by the different nature of neutron interactions that can occur within the material: the elemental composition within the growth volume can be studied through the neutron resonance absorption^7^,^8^,^9^,^10^,^11^,^12^ the temperature distribution can be measured through the Doppler broadening of neutron resonances^13^,^14^,^15^; some crystallographic properties can be investigated through the coherent neutron scattering^16^,^17^; and the shape and location of the solid/liquid interface, the presence of cracks, impurities, and macroscopic defects can be revealed by neutron transmission imaging and tomography^18^,^19^.

In this study, we demonstrate the ability of ***energy-resolved neutron imaging*** implemented at a pulsed neutron source to provide quantitative information on growth conditions and some crystal properties in real time (in crystal growth terms) and *in situ*. The measurements reported in this paper were performed during two solidification runs of the scintillator BaBrCl:Eu^2+^ (0.5 and 5 mole % Eu) in the Vertical Gradient Freeze (sub-type of the Bridgman) configuration.

## Results and Discussion

### Neutron resonance absorption imaging setup

**Figure 1:**
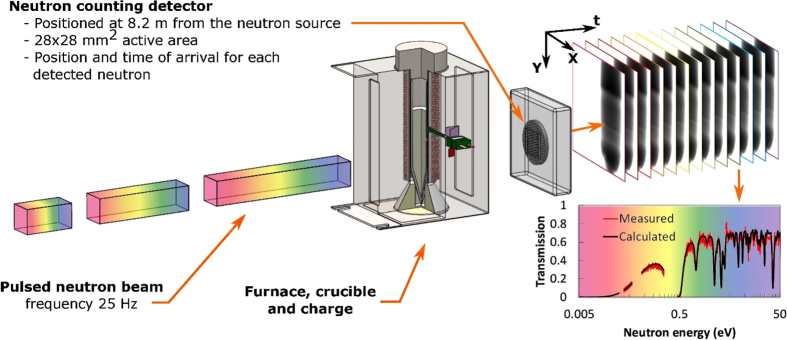
Schematic diagram of the experimental setup. A pulsed neutron beam travels towards the neutron counting detector installed at ~8.2 m from the source. Both position (~55 μm) and time (0.1–1 μs) are measured by the detector for each registered neutron. A furnace with a BaBrCl:Eu charge is installed a few centimeters from the detector. A set of images, each corresponding to a particular neutron energy, is acquired in each experiment, spanning neutron energies from epithermal range (1–100 eV) to cold neutrons of meV energies. 262,144 spectra are acquired simultaneously (within each of the 512 × 512 pixels of 55 × 55 μm^2^ area).

Measurement of neutron transmission spectra in a wide range of energies, including epithermal, thermal, and cold ranges, allows for a quantitative analysis of the elemental composition within the sample through the resonance absorption typically appearing at the epithermal neutron energies. At certain energies specific to a particular element (to a specific isotope of an element, to be more precise) neutron attenuation increases sharply, leading to sharp dips observed in the spectrum measured in the area where such element is present. These are the resonance features in the transmission spectrum (e.g., the spectrum shown in Fig. 1) which provide the possibility to measure the uniformity of distribution of a particular element within the growth volume in the experimental setup shown in Fig. 1. The depth of resonance absorption dip is determined by the amount of a particular element along the neutron propagation path. Therefore, not only the presence of an element can be recovered from our experiments, but also a quantified elemental composition can be obtained from the measured transmission spectra in each pixel of our data set. Not all elements/isotopes can be mapped with the present instrumentation, as some of them exhibit resonances only at very high neutron energies. Our spectroscopic neutron imaging is presently limited to the elements that exhibit substantially strong resonances at energies below ~10 keV^9^,^10^. For example, visualization of the liquid/solid interface during the growth of un-doped BaBrCl crystals will require much longer integration times as the contrast in that case will be provided only by the difference in the density between the two phases.

### Shape and location of the liquid/solid interface

**Figure 2:**
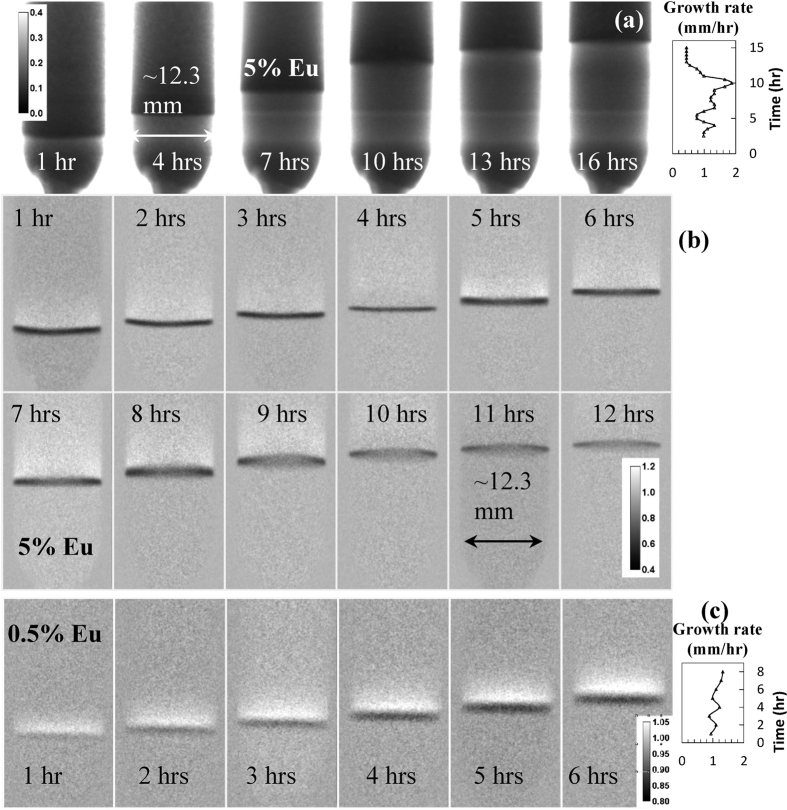
Location and shape of liquid/solid interface visualized at different stages of the crystal growth. (**a**) Neutron transmission images of BaBrCl:5%Eu sample during the crystal growth procedure. Images are integrated over 30 minutes and acquired for neutrons in the energy range of 9.76 meV – 31.10 meV. The time since the start of crystal growth is indicated in each image. A brighter horizontal line in the middle of the sample corresponds to a slower growth rate when furnace temperature was held nearly constant between hours 3 and 4. The calibration bar indicates the sample transmission. (**b**) The ratio of two consecutive images (30 min acquisition each) is shown to emphasize the location of interface between liquid and solid phases. The darker areas correspond to reduced Eu concentration relative to the previously acquired image as the Eu is rejected by the growing interface into the liquid by normal segregation. (**c**) – same as (**b**) except for the sample with 0.5 mole % Eu doping, 1 hour image integration time. The movies S1 and S2 in the online Supporting information show the dynamics of interface observed during crystal growth.

The shape and location of the liquid/solid interface are among the important diagnostic parameters, which can be used for process optimization^20^. These can be visualized in nearly real time by white spectrum neutron transmission images^19^ in cases where the variation of elemental composition and/or density provides a sufficient contrast. Results of our experiments demonstrate that the neutron transmission images can reveal the location of the interface as the crystals were solidified over a ~16-hour and a ~9-hour period for the 5 and 0.5 mole % Eu doped samples, respectively, Fig. 2a. The growth rate in these experiments was intentionally varied between 0.5 and 2 mm/hr in order to investigate the dependence of Eu segregation on the growth rate. The transmission images clearly indicate that Eu concentration in the resulting crystal is lower for slower growth rates, as Eu is rejected into the melt due to segregation^21^. More detailed information on the interface shape can be obtained by the differential imaging (Fig. 2b and c for 5 mole and 0.5 mole % Eu doping, respectively), where the ratios between the consecutive images are shown. The black line in these images is representative of the effective growth rate. The dynamic of interface location within the growth volume can be seen in the time-resolved movies S1 and S2 in the online Supplementary information. The images of Fig. 2 and these movies demonstrate, firstly, that we can image the interface during crystal growth, and secondly, that the shape of the interface is affected by subtle changes in the growth environment (such as the location of the insulation/heater vs. the interface location and the height of melt vs. solid) as the thermal profile of our furnace was not actively controlled. The shape of the interface in our experiment with the 5 mole % sample changed from concave during the first 3 hours of growth, to a nearly straight line (as observed between 4^th^ and 8^th^ hours) to convex as the growth proceeded.

The images of Fig. 2 also reveal the inhomogeneity of Eu concentration due to normal segregation. The build-up of a boundary layer ahead of the growth front is clearly observed indicating that the Eu activator has a segregation coefficient < 1. The sharp Eu concentration gradient across the solid-liquid interface, calculated previously analytically^22^,^23^, determines the concentration of Eu in the growing crystal, and its control is technologically essential. The visualization presented in this paper, to the best of our knowledge, has never been achieved *in situ* and in real time. A higher transmission of the boundary layer is observed as the growth rate is increased (e.g., at 5^th^ and 7^th^ hours, Fig. 2b).

### Mapping of the elemental composition

The presence of sharp variation of neutron attenuation at energies specific to a particular element (particular isotopes of element, to be more precise) can be used for the spatially-resolved analysis of elemental composition of the sample, as demonstrated in this section. The sensitivity of this technique obviously depends on the resonance attenuation cross sections, which for many elements exist in the epithermal range of neutron energies (~eV to ~keV neutrons), which matches the accessible range of energies for the present energy-resolved neutron imaging facilities. However, there are also elements which exhibit resonances only at higher energies (~MeV) which cannot be resolved with good spatial resolution, which makes their quantification nearly impossible for the methods described in this section. Therefore there are two parameters crucial for the applicability of this method: the value of resonance cross section should be sufficiently large (>10^2^–10^3^ barns) and the energy of this resonance should be less than ~10^3^ eV. These two parameters and the thickness of the sample determine the limits of elemental concentration which can be mapped by energy-resolved neutron imaging. Consequently, the possibility of elemental mapping for a particular element in a particular material can be accurately estimated from the tabulated cross sections^24^. Among the easily measurable elements are Sm, Cd, In, Eu, Hf, Ag, Au, Cs, W, Co, Br, Rh, Tm, U, Ir, Re, and many other elements, while C, O, Be, Mg, Ca, Si, Tl, N, P are among those elements which are almost undetectable^10^,^24^. One of the attractive features of this technique is the fact that even if some specific resonances of particular elements overlap (e.g. resonance of Ag and Au are very close to each other (around 5 eV), these elements can still be distinguished by the other resonances, which do not overlap. There it is important that the transmission spectrum can be measured in a wide range of energies. Although the elemental maps presented in this paper were measured for a specific material (BaBrCl:Eu scintillator) this method can be attractive for other studies where conventional techniques fails due to opacity of materials.

**Figure 3:**
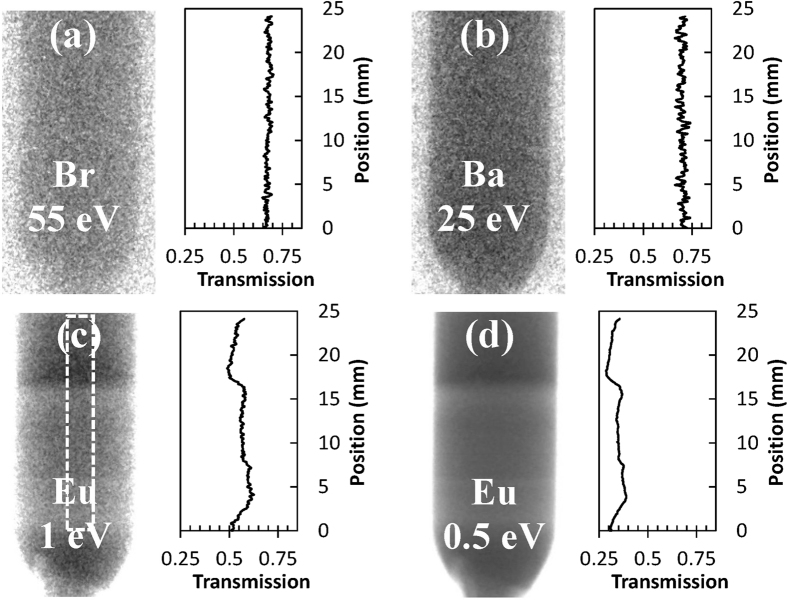
Element-specific neutron transmission images. Each image was acquired at resonance energy characteristic to Ba, Br, and Eu, respectively. All images are acquired in one measurement at the end of the growth procedure (corresponding to 16th hour in Fig. 2a; the top part of the sample is still in the liquid phase). Cross sections through the images are taken over the same area indicated by the dashed rectangle in Fig. 3c. Note that the cross sections start at the sample location where crystal growth started in our experiment (~5 mm from the bottom of the original charge). Resonance energies of the elements are shown in the legend of each image. No measurable variation of Ba or Br concentration was observed across the sample, while Eu exhibited distinct reduction of its concentration in the solid phase. The variations in Eu concentration in the solid part are due to changes in interface velocity.

**Figure 4:**
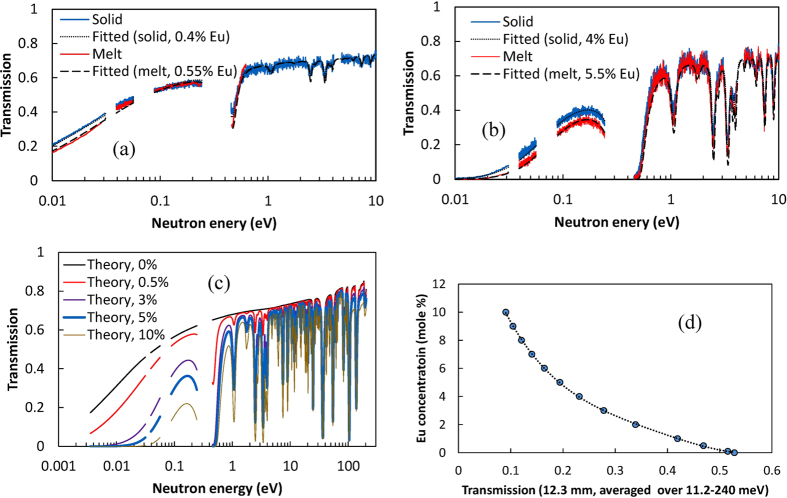
Neutron transmission spectra of BaBrCl:Eu material. (**a**,**b**) Typical measured (solid lines) and calculated (dashed lines) transmission of the BaBrCl:Eu sample of ~12.3 mm thickness with different levels of Eu doping: nominal doping of 0.5 mole % and 5 mole %, respectively. The calculated curves are obtained from the ideal transmission (shown in (**c**) for various levels of Eu doping, derived from the tabulated cross sections) convolved with the function describing the width of the neutron pulse. (**d**) BaBrCl:Eu transmission averaged over 11.2–240 meV neutron energies and calculated for a 12.3 mm sample. That curve is used for the spatially resolved reconstruction of Eu concentration shown in Fig. 5.

**Figure 5:**
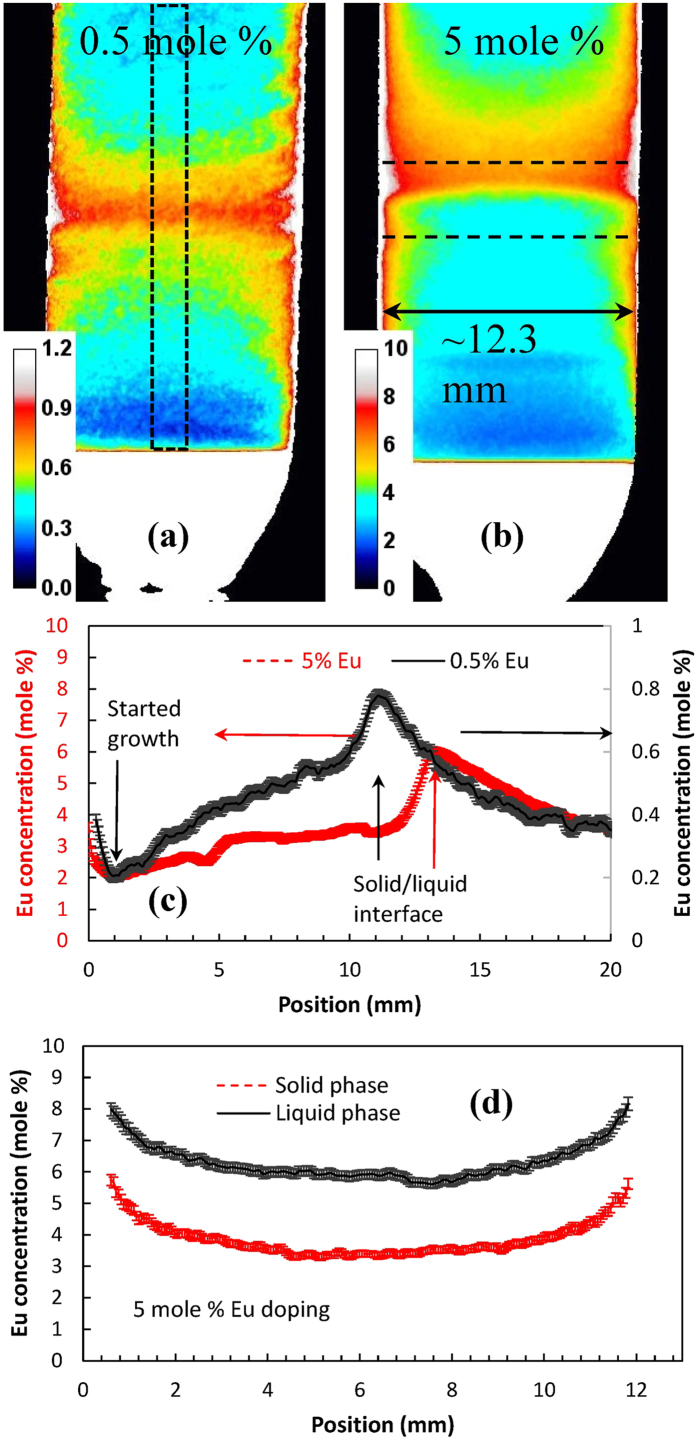
Map of Eu concentration reconstructed from measured data. (**a**,**b**) Map of Eu concentration within the ampule reconstructed from the measured neutron transmission over 7.12–31.10 meV energy range (for the 0.5% sample measured after 8 hours of crystal growth., (**a**)) and over 11.2–240 meV (for the 5% sample measured after 10 hours of growth (**b**)). The color scale indicates the reconstructed Eu concentration in mole %. (**c**) Cross section (~2.6 mm wide) through the reconstructed concentration maps indicated by a dashed rectangle in (a). (**d**) Horizontal cross section (~0.5 mm wide) through the reconstructed Eu concentration map of 5 mole % Eu sample, shown by dashed lines in (**b**). The accuracy of reconstructed Eu concentration values was ~0.127% and 0.04% for 5% and 0.5% Eu doping samples, respectively. The top part of both samples was in the liquid phase during these measurements. The increase of Eu concentration is clearly seen above the liquid/solid interface.

The element-specific images of Ba, Br, and Eu (Fig. 3) were reconstructed from the same experimental data (Cl does not have resonances in the accessible energy range). Cross sections through these images indicate that no measurable variation of Br and Ba concentration was observed within our samples. The concentration of Eu activator can be accurately reconstructed both in the liquid and solid phases due to the strong dependence of sample transmission on Eu concentration, as demonstrated in Fig. 4. In the crystal lattice of BaBrCl, europium replaces barium atoms. Therefore, in areas where the Eu concentration is increased, we should observe a reduction of Ba concentration. However, since Ba resonance attenuation is relatively weak, only the Eu distribution was quantified very accurately. The transmissions of a 12.3 mm thick BaBrCl:Eu sample calculated for various Eu concentrations shown in Fig. 4c were used to produce a calibration curve (transmission integrated in a given energy range vs. Eu concentration), shown in Fig. 4d. That curve was used for the reconstruction of Eu concentration from the measured transmission spectra. The results of this reconstruction, shown in Fig. 5, depict a quantitative map of Eu concentration within the ampule during the crystal growth process. To obtain these maps, the measured transmission spectrum within each pixel was matched to the transmission spectrum with a corresponding Eu concentration calculated from the tabulated cross sections of Ba, Br, Cl, and Eu isotopes with the assumption of their natural abundance (Fig. 4c and d). Since the thickness of the sample was known in each pixel of the image (sample occupies the known ampule volume) the fitting of simulated data into the experimental transmission was repeated for each 55 μm pixel of these images, allowing for an accurate mapping of Eu concentration within the ampule even for a 0.5 mole % doping level. The increase of Eu concentration just above the solid/liquid interface, seen for both the 0.5 and 5 mole % Eu doping samples (Fig. 5a,b), unambiguously explains the contrast seen in the white-spectrum transmission images of Fig. 2. This confirms that the contrast for thermal neutron radiography of BaBrCl:Eu samples is dominated by Eu.

**Figure 6:**
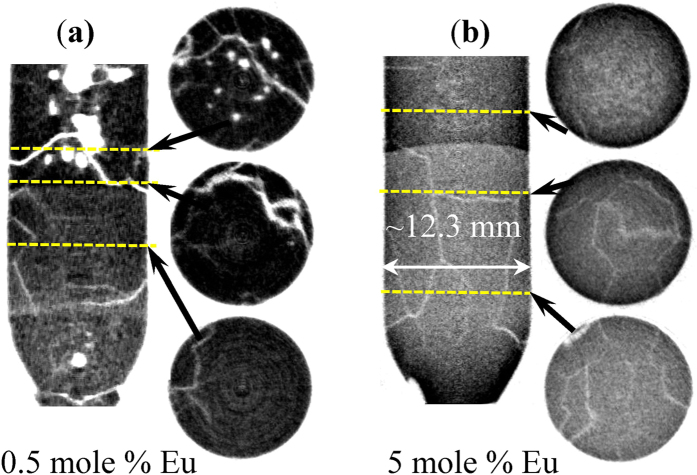
Tomographic reconstruction of BaBrCl:Eu ingots. (**a**) 0.5 mole % of Eu; (**b**) 5 mole % of Eu. Measurement was performed after the crystal growth by slow solidification was stopped and samples were rapidly cooled over a few hours to room temperature. The darker areas correspond to a large Eu concentration. Multiple cracks are seen within the samples, as well as clusters of Eu-deficient areas (in the 0.5% Eu sample) which were formed by rapid solidification propagating from the top of the sample. The images are integrated over the full neutron spectrum of FP5 beamline. The dashed lines indicate the location of horizontal cross sections shown to the right from the vertical cross section through the middle of the samples.

The vertical cross sections through the maps of Eu concentration (Fig. 5c) show that the average Eu concentration increases within the grown crystal as the crystallization proceeds. The boundary layer above the interface extends into the liquid by about 7 to 8 mm, which is indicative of low convective mixing in the melt. In the configuration used (melt height of about 4.5 cm) a quasi-steady state appears with incorporation of about 3.2% Eu (growth rate 2 mm/hr for the sample with a nominal melt concentration of 5%). A steady state is not achieved for the sample with a nominal melt concentration of 0.5%. In both cases, the data indicate an effective segregation coefficient of about 0.4, as calculated from the ratio of Eu concentration values at the beginning of growth and just above the solid/liquid interface (Fig. 5c). Some radial segregation of Eu was also observed, as seen from the horizontal cross sections shown in Fig. 5d. Obviously these cross section represent an average value of Eu concentration integrated through the thickness of the sample as seen by the incoming neutron flux. To properly quantify the distribution of Eu concentration in all 3 dimensions multiple projections have to be taken to allow tomographic reconstruction, as described in next section. However, the trend of increased Eu concentration towards the edges of the sample can still be observed from this single-projection measurement. This is also confirmed by the tomographic reconstruction of sample attenuation shown in Fig. 6.

We emphasize that these maps of Eu distribution are obtained *in situ*, while the crystallization proceeds. Such elemental quantification with ~100 μm spatial resolution requires relatively long integration times, on the scale of 0.5–1 hour, that are compatible with the time scale of crystal growth processes and therefore can still be implemented for *in situ* optimization of crystal growth parameters and provide valuable information for validation of simulations of the crystal growth processes.

### Tomographic imaging of crystal defects

Location of macroscopic defects, such as cracks, macroscale impurities ( > 100 μm), and variation of elemental composition, can also be visualized in three dimensions through tomographic reconstruction, which typically requires measuring a large number of transmission images while the sample is rotated along the axis perpendicular to the incoming neutron beam. In principle, that measurement can be performed during *in situ* growth in a Bridgman furnace equipped with both sample translation and a rotation stage. Not having that set-up available, we have conducted tomographic imaging after the crystals were grown and cooled rapidly to room temperature, Fig. 6. Upon rapid cooling and solidification of the leftover liquid, the formed crystals are subject to significant stress released by formation of a large number of cracks. The results of tomographic reconstruction (also shown in 3 dimensions by movies S3,S4,S5,S6,S7,S8 in online Supplementary information) indicate that many of the cracks originate at the areas where Eu concentration was substantially reduced compared with the neighboring areas, especially for the sample with 0.5 mole % Eu doping. The increased Eu concentration towards the ampule walls seen in Fig. 5d is also confirmed by that reconstruction, shown in Fig. 6.

## Conclusions

The *in situ* diagnostics technique demonstrated in this paper provide a new tool for an efficient guidance for the development of crystal growth processes for new materials. Obviously, the limited number of experimental facilities make the technique described in this paper unavailable for industrial scale crystal production. However, once computer simulations are fully validated by such experiment, scale-up of a particular crystal growth process, especially for new materials, can be performed from data acquired at the beamline. The applications of this technique are not limited to crystal growth of scintillator materials; it can easily be used toward development and optimization of many other material processes.

## Methods

The results of the presented study have been made possible by the use of i) the recently developed unique 2D fast neutron counting detector^25^,^26^ allowing for the spatially resolved measurement of neutron transmission spectra with energies ranging from thermal and cold ranges (10 meV to 0.5 eV) to epithermal neutrons (~0.5 eV to several keV); and ii) spallation neutron sources (e.g. those described in references^27^,^28^,^29^,^30^), where neutron transmission in a wide range of energies can be measured simultaneously through the time-of-flight (TOF) technique^31^,^32^ as well as at continuous sources with the use of crystal monochromators, which are limited to only thermal and cold neutrons^33^.

**Figure 7:**
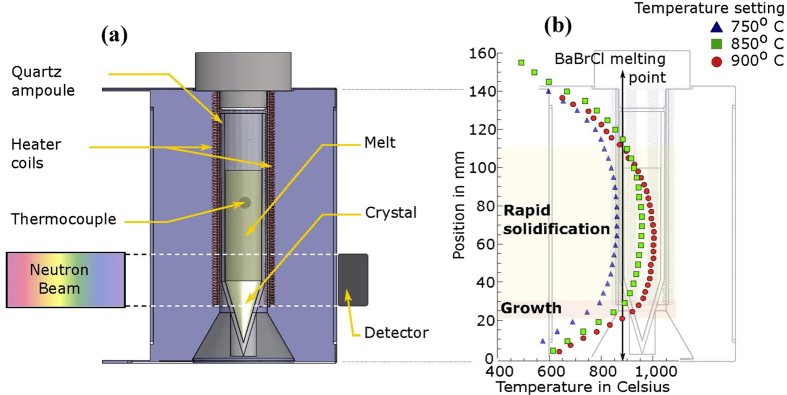
Furnace used in the experiment. (**a**) Schematic diagram of the furnace used for the *in situ* experiments. (**b**) Temperature profile within the furnace measured for three different nominal temperature settings of 750, 850, and 900 °C. The melting point of BaBrCl at the start of the experiment is located within the temperature gradient and moves up when the control temperature is reduced.

The proof-of-principle experiments on *in situ* diagnostics of crystal growth in a Bridgman furnace were conducted at a pulsed neutron beamline FP5^34^ at the Los Alamos National Laboratory (Fig. 1). A compact furnace was designed specifically for this experiment allowing a short distance between the sample and the detector to avoid image blurring by the finite neutron beam divergence, which was ~0.18 degrees in our experiment. The neutron counting detector with a 28 × 28 mm^2^ active area^25^,^26^ recorded position and time of arrival for each detected neutron, enabling simultaneous measurement of the neutron transmission spectrum in each 55 × 55 μm^2^ pixel within an energy range of ~0.005–100 eV. Two charges of BaBrCl:Eu with 0.5 mole % and 5 mole % Eu doping were sealed under vacuum before the experiment into ~12.3 mm ∅ silica glass ampules. This material^35^ is opaque to conventional X-ray techniques. The ampules were placed in the center of the clam-shell furnace with a single heating element (Fig. 7a). In future experiments, we will implement control of the thermal environment in a multi-zone furnace supported by computer simulations and attempt to maintain the preferred concave shape of the interface through the entire process.

The temperature gradient within the furnace was calibrated prior to the measurements (Fig. 7b). The BaBrCl:Eu crystals were grown from the melt by gradually lowering the temperature of the furnace. The growth of these crystals was far from ideal as these experiments were aimed at the demonstration of possibilities for *in situ* diagnostics, rather than growing best quality crystals. In particular, the initial polycrystalline charge was not melted to the bottom of the ampoule. This did not allow proper nucleation of the crystals and growth of single crystals, but was necessary to test the *in situ* imaging starting on a defined stabilized interface. All images presented in this paper were normalized by the images taken with no sample present in the beam to eliminate the non-uniformities of the neutron beam, detector response and the image features produced by the attenuation by the furnace. An accurate selection of materials with low neutron attenuation cross section was performed before the furnace was built in order to minimize neutron attenuation in the furnace and crucible. Most heat insulating ceramics and quartz crucibles have very low neutron attenuation and the most opaque elements of our experimental setup were thin wires of the heating elements. All the transmission spectra were also corrected pixel by pixel for the spectrum of the incoming neutron beam, background contribution, and for the transmission of the furnace and the crucible.

## Additional Information

**How to cite this article**: Tremsin, A. S. *et al*. Real-time Crystal Growth Visualization and Quantification by Energy-Resolved Neutron Imaging. *Sci. Rep.* **7**, 46275; doi: 10.1038/srep46275 (2017).

**Publisher's note:** Springer Nature remains neutral with regard to jurisdictional claims in published maps and institutional affiliations.

## Supplementary Material

Supplementary Information

Supplementary Movie S1

Supplementary Movie S2

Supplementary Movie S3

Supplementary Movie S4

Supplementary Movie S5

Supplementary Movie S6

Supplementary Movie S7

Supplementary Movie S8
