# Supplementary material for: Real-time Crystal Growth Visualization and Quantification by Energy-Resolved Neutron Imaging
Source: Sci Rep. 2017 Apr 20;7:46275. doi: 10.1038/srep46275 (PMC5397867; doi:10.1038/srep46275)

**Title: Real-time Crystal Growth Visualization and Quantification by Energy-Resolved Neutron Imaging**

**Authors:** Anton S. Tremsin, Didier Perrodin, Adrian S. Losko, Sven C. Vogel, Mark A.M. Bourke, Gregory A. Bizarri, Edith D. Bourret

# Supplementary Information

The scans through the entire sample volume (Movies S1-S8) give a clearer view on the internal structure of BaBrCl:Eu samples grown in our experiments. The interface between the grown crystal and the volume quenched from the liquid phase is clearly seen in these movies, confirming the results obtained in 2-dimensional projected imaging shown in Figs. 2, 3, and 5, which were measured during the crystal growth procedure. The convex shape and sharpness of the interface for the 5 mole % sample is visible due to the substantial change in Eu concentration across that interface boundary, with Eu expelled from the solid to liquid phase. During the rapid cooling process, a large number of mm-scale Eu-deficient clusters formed in the 0.5 mole % Eu doping sample. The tomographic reconstruction also confirms the increase of Eu concentration towards the periphery of both the grown crystals and the quenched part of the samples. In future experiments we will investigate at what temperature during the sample cooling these cracks are forming and attempt to correlate temperature with the dopant segregation process.

**Movie S1.**

Time evolution of interface between liquid and solid phase during crystal growth over ~16 hour period. BaBrCl:Eu with 5 mole % Eu doping. The interface is visualized by the ratio of two consecutive images, acquired over 30 minute periods. The contrast is mainly due to the variation

of Eu concentration between the solid and liquid phases – Eu is rejected at the solid-liquid interface due to normal segregation. The time since the beginning of growth is shown in the right corner. The last image in that sequence is taken after the sample was cooled down to room temperature. Shrinking of the sample leads to the formation of a dark edge at the sample periphery.

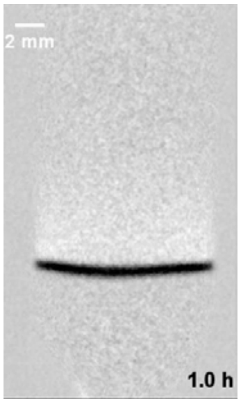


**Movie S2.**

Time evolution of interface between liquid and solid phase during crystal growth over ~9 hour period. BaBrCl:Eu with 0.5 mole % Eu doping. The interface is visualized by the ratio of two consecutive images, acquired over 60 minute periods. The contrast is mainly due to the variation of Eu concentration between the solid and liquid phases – Eu is rejected at the solid-liquid interface due to normal segregation. The time since the beginning of growth is shown in the right corner.

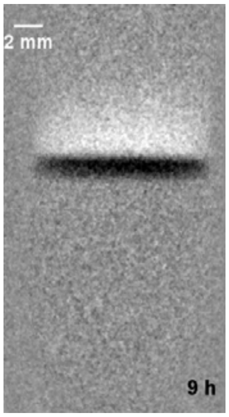

**Movie S3.**

Tomographic reconstruction of BaBrCl:Eu sample with 5 mole % Eu doping, measured after the sample was cooled down to room temperature. Scan through the vertical slices across the sample is shown, visualizing the location of multiple cracks formed during rapid cooling from ~800 C to room temperature. The concave shape of the interface between the liquid and solid phases present just before the rapid solidification is clearly visible. That interface is averaged over the sample thickness in Fig. 2 and in the movie S1, resulting in the apparent blur of the interface shape.

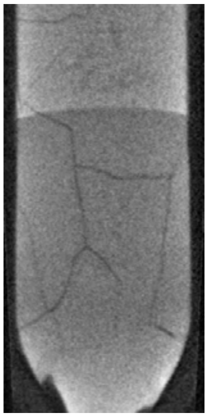

**Movie S4.**

Same as Movie S3, except scanned through the vertical axis of the sample.

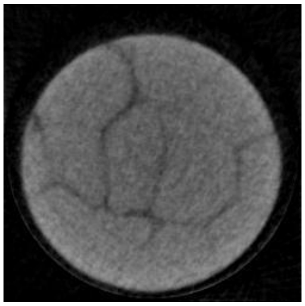

**Movie S5.**

A 3 dimensional view of the tomographic reconstruction of BaBrCl:Eu sample with 5 mole % Eu doping.

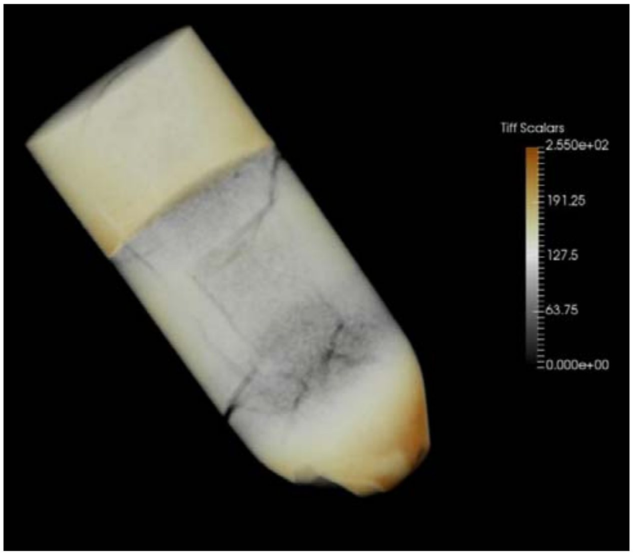


**Movie S6.**

Same as Movie S3, except measured with 0.5 mole % Eu doping sample. The dark areas in the slices correspond to location of cracks (lines across the sample) as well as Eu-deficient area (typically in a round shape).

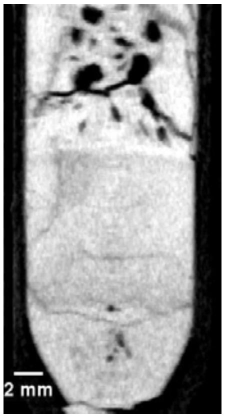


**Movie S7.**

Same as Movie S4, except measured with 0.5 mole % Eu doping sample. The dark areas in the slices correspond to location of cracks (lines across the sample) as well as Eu-deficient area (typically in a round shape).

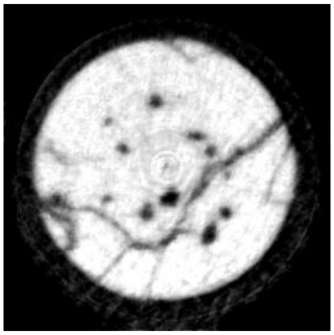

**Movie S8.**

Same as Movie S5, except measured with 0.5 mole % Eu doping sample.

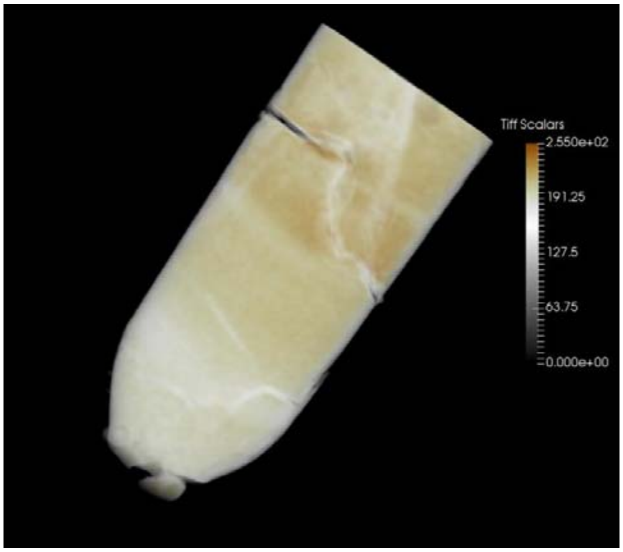

Supplement: Supplementary Information [file srep46275-s1.pdf]
